# Emotional intelligence and depressive symptoms in Spanish institutionalized elders: does emotional self-efficacy act as a mediator?

**DOI:** 10.7717/peerj.2246

**Published:** 2016-07-21

**Authors:** Octavio Luque-Reca, José María Augusto-Landa, Manuel Pulido-Martos

**Affiliations:** Faculty of Humanities and Education Sciences, Department of Psychology, University of Jaén, Jaén,Spain

**Keywords:** Depression, Emotional intelligence, Emotional self-efficacy, Institutionalized older adults, Mental health

## Abstract

**Background.** This work examines the relationship between emotional intelligence (EI) and depressive symptomatology in institutionalized older adults, delving into the mechanisms underlying this relationship. Considering that previous evidence of the variation of the EI-depression relationship depending on whether the emotional ability or the perception of that ability is evaluated, a model of multiple mediation was tested in which the dimensions of emotional self-efficacy (ESE) act as mediators in the relationship between ability EI and depressive symptomatology.

**Methods.** The sample consisted of 115 institutionalized older adults (47.82% women; 80.3 ± 7.9 years of age) from the province of Jaén (Spain) who completed a test of ESE, a measure of ability EI, and a self-administered questionnaire of depressive symptoms.

**Results.** The results showed a positive association between older adults’ emotional performance and depressive symptomatology, finding stronger associations with ESE than with EI abilities. In addition, multiple mediation analyses showed that two of the four dimensions of ESE fully mediated the relationship between ability EI and depressive symptoms.

**Discussion.** These findings suggest that older adults’ high levels of emotional competence generate a feeling of ESE which can protect them against depressive symptoms. This work supports the predictive validity of emotional abilities and ESE for the mental health of a group that is particularly vulnerable to depression, institutionalized older adults. The limitations of the work are discussed, and future lines of research were considered.

## Introduction

Depression is a major and prevalent problem in the population (Moussavi et al., 2007), which has a strong impact on quality of life (Swan, Watson & Nathan, 2009). In addition to the resulting distress and incapacitation, depressive disorders are associated with reduced life expectancy (Ensinck et al., 2002). For decades, numerous studies have tried to determine whether depression and its symptoms are more predominant in some age groups than in others. Clearly inconsistent data have been found concerning the issue of whether older adults have increased vulnerability to depressive problems than younger adults (Snowdon, 2003). What does seem clear is that depressive disorders, which would include both depressive symptoms and clinical depressive disorders, are a problem of particular relevance in the field of gerontology (Riquelme, Buendía & López, 2006). In addition, the scientific literature has found certain peculiarities of the disorder in older adults, in particular, a higher proportion of physical symptoms and a lesser feeling of sadness than in younger people (Baldwin, 2008), as well as higher relapse rates (Mitchell & Subramaniam, 2005).

Within the group of older adults, greater vulnerability to depression has been observed in those who are institutionalized (Jongenelis et al., 2004; Ron, 2004; Boorsma et al., 2012). In particular, a review of studies (Djernes, 2006) points out that, in Europe, generally between 10 and 25% of older adults residing in their own home experience depressive symptoms compared to a percentage ranging from 32 to 48% in institutionalized people. These data, together with those that emphasize the upward trend in the number of institutionalized older adults in countries such as Spain (Instituto Nacional de Estadística, 2004; Instituto Nacional de Estadística, 2013), make it essential to delve into the factors that determine the mental health of this priority population group. Thus, both reviews (Djernes, 2006) and studies specifically focused on institutionalized older adults (Santiago & Mattos, 2014) agree that among the leading determinants of depressive disorders are mainly demographic, social, psychological, functional and health aspects. However, while some of these variables (i.e., comorbidity with other diseases, pain, cognitive impairment, previous hospitalizations, lack of social support, etc.) have been widely studied and associated with the prevalence of depression in institutionalized older adults (McCusker et al., 2013; Santiago & Mattos, 2014), others, such as the aspects related to the management of emotions, have received scarce attention to date (Lloyd et al., 2012). Therefore it is necessary to identify those variables that can promote and have a positive impact on the wellbeing of this group.

Among these emotional factors, the construct of emotional intelligence (EI), which is a nexus between the psychological processes of cognition and emotion (Jordan, Troth & Ashkanasy, 2013), has been shown to promote a more adaptive functioning of human beings (Schutte & Malouff, 2013). In fact, several meta-analysis confirm that EI is positively associated with mental health (Schutte et al., 2007; Martins, Ramalho & Morin, 2010) and can predict an individual’s greater psychological adjustment (Mayer, Roberts & Barsade, 2008). Although there are various theoretical conceptualizations of the construct, the ability model of Salovey & Mayer (1990) and Mayer & Salovey (1997), which considers EI as a series of skills or mental abilities related to emotional information processing, is the most widely accepted and used by the specialized scientific community (Mayer, Roberts & Barsade, 2008). From this conceptualization, EI is defined as “the ability to monitor one’s own and others’ feelings and emotions, to discriminate among them, and to use this information to guide one’s thinking and action” (Salovey & Mayer, 1990, p. 189). EI is made up of four key emotional abilities: (1) accurate perception, appraisal, and expression of emotions; (2) access to and/or generation of feelings that facilitate thought; (3) understanding of emotions and generation of emotional knowledge; and (4) regulation of emotions by promoting emotional and intellectual growth (Mayer & Salovey, 1997).

Two types of instruments have been used to assess EI: measures of ability, which measure the level of EI that the subject displays in a series of exercises and activities of emotional content; and self-report measures, which are those that assess emotional capacity as estimated by the individual. According to some authors, in the latter case, these self-perceptions of emotional functioning are primarily measuring a person’s belief in his/her emotional ability (Kirk, Schutte & Hine, 2008). Within the framework of the theory of efficacy beliefs, Bandura (1977) states that the degree of self-efficacy experienced in a specific field of functioning conditions the results that people expect to obtain through their efforts, thus affecting their actions and life achievements. Thus, self-efficacy beliefs also influence the amount of effort that people invest in coping with difficult situations and their vulnerability to stress and depression (Caprara et al., 2008). In this regard, self-efficacy may be essential to explain various psychological phenomena of the human being. We also note that self-efficacy is not a global construct, but a set of specific subconstructs of self-efficacy related to different fields of functioning (Bandura, 2006). Within the emotional area, as an example of a subconstruct of self-efficacy, the concept of regulatory emotional self-efficacy (RESE) is introduced (Bandura et al., 2003), which refers to “beliefs in one’s capability to ameliorate negative emotional states and to experience positive emotions” (Choi, Kluemper & Sauley, 2013, p. 99). In fact, a negative association between RESE and incidence of depressive disorders has been established (Caprara et al., 2003). Another subconstruct of self-efficacy, which encompasses the perception of a greater number of emotional abilities than RESE and which is based on the ability model of EI (Mayer & Salovey, 1997), is emotional self-efficacy (ESE). ESE refers to people’s self-perception of their emotional ability, that is, their competence to perceive, use, comprehend, and regulate their emotions; and it has shown positive associations with life satisfaction and rationale coping, and negative associations with stress and avoidance coping (Choi, Kluemper & Sauley, 2013). ESE would explain psychological and socially maladaptive results (Bandura, 1997) even if the person has the necessary skills for reason about their emotions and to use their emotions in facilitating thinking (Mayer & Salovey, 1997). It would act as a mediator in the relationship between real skill (ability EI) and actions or achieved results (Bandura, Adams & Beyer, 1977).

In the case of depression, several works have addressed the study of the EI-depression relationship, finding results of different magnitudes depending on the considered construct (ESE vs. ability EI). Thus, studies evaluating ESE (Fernández-Berrocal et al., 2005; Extremera et al., 2006; Goldenberg, Matheson & Mantler, 2006; Williams et al., 2009; Lloyd et al., 2012) tend to find a stronger inverse relationship with depression or its symptoms than works evaluating ability EI (Extremera et al., 2006; Goldenberg, Matheson & Mantler, 2006; Williams et al., 2009). However, in both cases, the person’s emotional abilities, either real or perceived, contribute significantly to experiencing lower levels of depressive symptoms.

Previous research has analyzed the moderating role of the ESE in the relationship between ability EI and depressive symptoms (Salguero et al., 2015), but this is not enough in explaining the relationship between these variables. In this sense, it is essential to move towards causal models that add “how” certain effects occur rather than “when” these effects occur (Baron & Kenny, 1986; Frazier, Tix & Barron, 2004). Given a sufficiently demonstrated relationship between variables, when the role of third variables is analyzed, the aim is to reach the mechanism explaining the association and this is an example of maturity of any field of inquiry (Hoyle & Kenny, 1999). An example of this progress is the analysis of the mediating role of self-efficacy in the effect that social support has on depression (Cutrona & Troutman, 1986; Saltzman & Holahan, 2002).

Therefore, with this work, we proposed as an explanatory hypothesis that ESE will be the key variable in the mechanism underlying the positive relationship between ability EI and the level of depressive symptoms. Specifically, high levels of ability EI would ensure more frequent mastery experiences, one of the antecedents of general self-efficacy (Bandura, 1977; Bandura, 1986; Bandura, 1997), due to the success in coping with emotional tasks, and this may also allow increased levels of ESE. Likewise, people with high levels of ability EI would have less emotional arousal when they have to deal with negative situations (Conger & Kanungo, 1988), which could also lead to increased levels of ESE. In fact, the levels of ESE are variable and can be improved through personal experiences (Caprara et al., 2008; Kirk, Schutte & Hine, 2008). In the proposed mediational model, the levels of ability EI would ensure both the success in coping with emotional tasks and a lower experimentation of negative emotions, thus promoting higher efficacy beliefs (Conger & Kanungo, 1988; Wise & Trunnell, 2001). Hence, as self-efficacy in a specific area has the capacity to determine an individual’s behavior in that area (Kirk, Schutte & Hine, 2008), people with higher levels of ability EI will probably have higher ESE, which will help to protect them from depression and its symptoms. In particular, it has been proposed that feeling ineffective (whether or not it is true) leads to an emotional activation that generates anxiety, fear, and apprehension, which negatively affect a person’s effort and resilience in the face of stressful or unpleasant situations (Bandura, 1997), which in turn could foment greater depressive symptomatology. On another hand, it would be logical for high ESE to generate feelings of trust and competence, enabling the person to deal with emotionally demanding situations with more self-assurance, effort, and efficiency. In this sense, and considering the study group, it is likely that older adults with high EI will consider themselves to be capable of perceiving, using, and regulating their emotions adequately, and that their confidence to manage their emotions will make them more competent to face the emotional discomfort associated with their everyday problems.

Moreover, given the population group in question, institutionalized older adults, it is important to explore the evolution that emotional beliefs and skills experience with age. Thus, in studies involving adults over 65, although both ESE and ability EI show positive associations with age (Kafetsios, 2004; Fariselli, Ghini & Freedman, 2006; Brasseur et al., 2013), when this issue is examined at quite advanced ages the results are less consistent. Specifically, whereas some dimensions of ESE and ability EI are negatively affected by age, others are increased over time (Fernández-Berrocal et al., 2012; Cabello et al., 2014; Fantini-Hauwel & Mikolajczak, 2014). These findings are in line with the idea that emotional processing capacity is maintained at older ages (Mikels et al., 2010), suggesting that some aspects of emotional functioning that are even increased throughout the lifespan (Samanez-Larkin & Carstensen, 2011). Therefore, the relatively low impact of age suggests that it may be interesting to dedicate more attention to the emotional functioning of the institutionalized older adults.

Given the above, the goal of the present study was to verify in a group of institutionalized older adults the existence of a multiple mediation model in which the dimensions of ESE fully mediate the documented relationship between ability EI and depressive symptoms. More specifically, as the first working hypothesis, we proposed that depressive symptoms will be positively associated with ESE and with EI evaluated as an ability, and that stronger correlations with ESE than with ability EI will be found (Extremera et al., 2006; Goldenberg, Matheson & Mantler, 2006; Williams et al., 2009). At the same time, and in line with previous works with different samples (Brackett & Mayer, 2003; Brackett et al., 2006), we expected to find low to moderate correlations between ability EI and older adults’ ESE (Caprara et al., 2008). As a second hypothesis, based on previous research that confirm the relationship between certain ESE dimensions and psychological adjustment variables (Choi, Kluemper & Sauley, 2013), we expected to find that the intrapersonal dimensions of ESE (self-emotional appraisal, use of emotion, and regulation of emotion) will fully mediate the inverse relationship between EI and levels of depressive symptomatology of institutionalized older adults.

## Materials & Methods

### Participants and procedure

The sample was made up of 115 adults over 65 (60 men and 55 women) institutionalized in residences of the province of Jaén (Spain), aged between 66 and 101 years (*M* = 80.33, *SD* = 7.95). The inclusion criterion was level of cognitive impairment, selecting only participants who were classified by their scores as ”without cognitive impairment” or ”with very mild cognitive impairment”. This excluded 67% of the residents from participating. Participants were informed of the purpose of the investigation and all gave their consent to participate in the study. The tests were administrated individually to ensure that the participants understood them. Before performing the study, it was approved by the Provincial Delegation of Jaén of the Ministry of Equality and Social Welfare of the *Junta de Andalucía*. It should be noted that, according to the general standards document of the Ethics Committee of the University of Jaén, it is not compulsory to apply for the approval of such committee when research is conducted in adults, using non-clinical questionnaires, and in non-health centres (i.e., residences for older adults), being the reason why this report has not been requested in this investigation. However, the study was conducted following the guidelines of the Declaration of Helsinki (59th General Assembly of the World Medical Association, Seoul, October 2008) and current Spanish legislation governing research on human subjects (Royal Decree 561/1993 on clinical trials).

### Instruments

*Mini-Mental State Examination* (MMSE; Folstein, Folstein & McHugh, 1975; Spanish version of Lobo et al., 1979). This 35-item tool evaluates an individual’s degree of cognitive impairment. Each hit is scored with a point, with the test score ranging between 0 and 35 points. As a cut-off point, we used the usual score of 24 points; overall score equal to or less than this figure indicates the existence of cognitive impairment. The Spanish version includes five more items than the original and is somewhat simpler. Hence, the cut-off point of 24 points in the Spanish version is considered equivalent to a 20-point score in the original version (Vinyoles et al., 2002). The test has shown adequate sensitivity and specificity for the detection of dementia (Lobo et al., 1979), as well as high test-retest reliability (Tombaugh & McIntyre, 1992).

*Mayer-Salovey-Caruso Emotional Intelligence Test* (MSCEIT; Mayer, Salovey & Caruso, 2002; Spanish adaptation of Extremera & Fernández-Berrocal, 2009). This 141-item instrument measures ability EI through performance in different tasks and emotional problems. This test is considered the best known and widely used measure of EI evaluated as ability (Fiori et al., 2014). In addition to evaluating the four dimensions of EI, it provides a global EI score which will be used for the purpose of this work. The test score ranges between 50 and 150 points. In the study carried out by Extremera, Fernández-Berrocal & Salovey (2006), the total reliability of the test was .94 for the scoring method based on experts and .95 for that based on consensus, and we used the latter criterion. As for the different dimensions, the reliabilities for the consensus score ranged between .82 and .93.

*Riquelme Depressive Symptoms Questionnaire* (Riquelme, Buendía & López, 2006). This instrument evaluates depressive symptomatology in gerontological population through 21 items that refer to the main symptoms of depression listed in the clinical criteria of the *Diagnostic and Statistical Manual of Mental Disorders* (American Psychiatric Association, 2000) and the *International Classification of Diseases* (World Health Organization, 1992) systems. It is rated on a 4-point Likert-type response scale ranging from 1 (*never*) to 4 (*most of the time*), with the total score ranging between 21 and 84 points. It has a high internal consistency, with an alpha of .91.

*Wong and Law Emotional Intelligence Scale* (WLEIS; Wong & Law, 2002; Spanish translation of Fernández-Berrocal et al., 2004). This 16-item instrument, which measures ESE (Ashkanasy & Dasborough, 2015), is rated on a 7-point Likert scale ranging from 1 (*strongly disagree*) to 7 (*strongly agree*). The test measures four dimensions: self-emotional appraisal (SEA); others’ emotional appraisal (OEA); use of emotion (UOE); and regulation of emotion (ROE). The dimensions SEA, UOE, and ROE assess intrapersonal aspects of ESE, while OEA evaluates interpersonal aspects. The score ranges between 4 and 28 points for each of these ESE dimensions. The scale also provides a global score of ESE, although for this work, we did not use it. The scale has adequate internal consistency indices, ranging between .83 and .90 (Wong & Law, 2002).

## Results

### Descriptive and correlational analyses

In addition to the descriptive statistics and internal consistency of the scales, Table 1 presents the results of the bivariate correlation analysis that shows the associations between ability EI, the dimensions of ESE, and older adults’ depressive symptoms. As expected, the level of depressive symptoms was statistically, significantly, and inversely associated with ability EI and ESE. Specifically, the ability EI-depressive symptoms correlation was smaller (−.19) than the correlation established between the ESE dimensions and depressive symptomatology (−.33, −.19, −.35, and −.31 for SEA, OEA, UOE, and ROE, respectively). However, as usual in the literature, we found a significant moderate association between institutionalized older adults’ global ability EI and the four dimensions of ESE (between .25 and .36).

**Table 1:** Descriptive statistics, internal consistency, and relationship between the variables of the study.

	1	2	3	4	5	6	M	SD	*α*
1. Overall ability EI	–						90.31	17.21	^a^
2. SEA	.26^**^	–					20.41	3.91	.79
3. OEA	.36^**^	.46^**^	–				18.99	5.07	.82
4. UOE	.33^**^	.39^**^	.45^**^	–			19.83	4.46	.82
5. ROE	.25^**^	.35^**^	.29^**^	.30^**^	–		20.12	4.86	.89
6. Depressive symptoms	−.19^*^	−.33^**^	−.19^*^	−.35^**^	−.31^**^	–	35.70	7.99	.85

**Notes.**

* *p* < .05.

** *p* < .01.

a could not be calculated for these dimensions because the instrument is scored via an Internet platform and the owner of the software does not allow access to raw scores of items. SEA, self-emotional appraisal; OEA, others’ emotional appraisal; UOE, use of emotion; ROE, regulation of emotion

### Multiple mediational analyses

**Figure 1:**
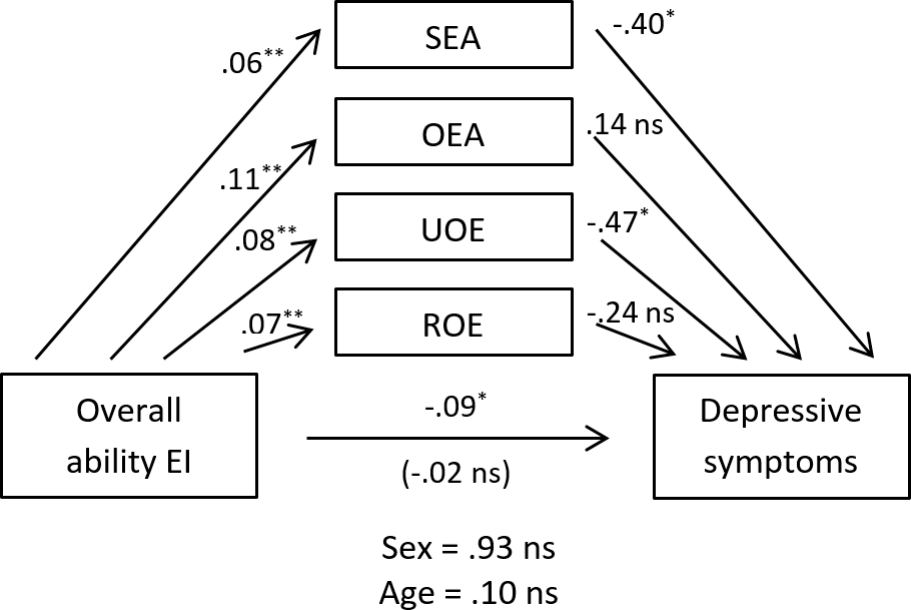
Multiple mediation model of the dimensions of ESE on the relationship of ability EI with depressive symptomatology, controlling for age and sex. The displayed values are non-standardized regression coefficients that estimate the strength of the relationship between the variables. SEA, self-emotional appraisal; OEA, others’ emotional appraisal; UOE, use of emotion; ROE, regulation of emotion.

Different multiple mediation analyses were conducted to explore whether the relationship between ability EI and depressive symptomatology is mediated by institutionalized older adults’ ESE. Considering the small size of the sample, we decided to follow the recommendations suggested by MacKinnon, Lockwood, & Williams (2004) and use the nonparametric method 5,000 repetitions to verify the significance of the proposed mediational model. With this procedure, more than one mediator can be analyzed simultaneously, and the possible influence of covariates on the model can be controlled. Thus, using the macros of Preacher & Hayes (2004) for IBM SPSS Statistics, we tested a multiple mediation model with four mediators (SEA, OEA, UOE, and ROE) and two covariates (sex and age), finding the results described below (see Fig. 1).

When analyzing the mediational effect of the four dimensions of ESE on the ability EI-depressive symptoms relationship, we found that the indirect effect of SEA was between −.073 and −.002, and the indirect effect of UOE was between −.090 and −.008, at a 95% confidence level in both cases. Thus, as 0 was not contained in any of the two previous confidence intervals, the indirect effect of SEA and UOE were both considered significantly different from 0 (*p* <.05), with both dimensions mediating the ability EI-depressive symptoms relationship. In addition, given that when the effect of the mediating variables was taken into account, ability EI ceased to have a significant impact on depressive symptoms, both dimensions of ESE exerted total mediation in relationship. As for the other two dimensions of ESE, OEA and ROE, multiple mediation analyses showed that their indirect effects on depressive symptoms did not reach significance, finding the value 0 between −.029 and .058, and between −.057 and .006, at a 95% confidence level. Therefore, the absence of mediation of both these dimensions in the ability EI-depressive symptoms relationship was assumed. It must also be noted that neither of the covariates controlled in the model (age and sex) significantly affected the ability EI-depressive symptoms relationship. Globally, the mediational model obtained explained 16% of the variance of institutionalized older adults’ depressive symptomatology, *F*(7, 107) = 4.10, *p* < .01.

## Discussion

The goal of the present study was to examine the relationship between emotional abilities and levels of depressive symptoms in a sample of institutionalized older adults, exploring at the same time whether older adults’ ESE acts as a mediator in that relationship and whether it can determine these individuals’ depressive symptoms.

The presence of depression in the older adults seriously affects their quality of life and is associated with a loss of individual functional capacity (Katon et al., 2003). It has an especially detrimental effect on health when it is comorbid with other diseases (Moussavi et al., 2007). Moreover, such comorbidity with other health problems may lead to underestimating the incidence of depression in people over 65 years of age, as some depressive symptoms may be confused or masked (Segulin & Deponte, 2007). Therefore, as institutionalized older adults are a collective with a high incidence of depressive symptoms (Djernes, 2006, for a review) and with lower levels of quality of life than noninstitutionalized older adults (Scocco, Rapattoni & Fantoni, 2006), it is vital to identify personal variables that can predict the onset of depression and protect residents from its significant negative consequences. In fact, depression has been revealed as a particularly important problem in residences for older adults (McCusker et al., 2013), and it should be considered and addressed by society as a whole.

Whereas EI has proven to be a good predictor of psychological adjustment (Mayer, Roberts & Barsade, 2008; Martins, Ramalho & Morin, 2010), so far, there is only one empirical work that has studied and confirmed the EI-depression relationship in older adults (Lloyd et al., 2012). Despite being a pioneer work, it exclusively analyzes older adults’ general emotional efficacy, without exploring its specific dimensions or emotional capacity assessed as an ability. In fact, evaluating EI through ability measures, like MSCEIT (Mayer, Salovey & Caruso, 2002), can minimize social desirability and other response biases (Lopes, Salovey & Straus, 2003). For those reasons, and considering institutionalized older adults’ increased vulnerability to depressive problems (Jongenelis et al., 2004; Ron, 2004; Boorsma et al., 2012), the findings from this study are especially important, extending for the first time the findings from other samples and age groups to institutionalized adults over 65 years of age. In general, the results confirm the relevance of emotional functioning—either via the individuals’ real ability or via their estimated degree of ESE—for the level of depressive symptoms experienced by residents.

Regarding the first working hypothesis, the results of the correlation analyses showed how depressive symptoms established stronger negative associations with the dimensions of ESE than with EI assessed as an ability, confirming the proposed hypothesis. Thus, like in other works using both types of measures of EI (Extremera et al., 2006; Goldenberg, Matheson & Mantler, 2006; Williams et al., 2009), a closer association between ESE and depressive symptoms in older adults is confirmed. According to some authors (Extremera et al., 2006), these low to moderate associations are expected because relationships with criterion variables like depression should not have a very narrow range, because being emotionally intelligent does not mean a person is always cheerful or forever seeking positive emotions (Cobb & Mayer, 2000). On another hand, and as expected (Brackett & Mayer, 2003; Brackett et al., 2006), the results also found moderate associations between ESE and ability EI, supporting the idea that both constructs cover different aspects of an individual’s emotional functioning (Fernández-Berrocal & Extremera, 2009). These results are in line with those obtained by Caprara et al. (2008), who find association coefficients around .30.

In relation to the second hypothesis, the results of the multiple mediation analysis indicated that ability EI had an indirect relationship with older adults’ levels of depressive symptomatology, mediated by certain ESE dimensions. Thus, in line with the idea that, when facing threatening events, self-efficacy beliefs can influence the emotional level (Bandura, 2001) and determine individual behavior (Kirk, Schutte & Hine, 2008), two of the four dimensions of ESE (SEA and UOE) predicted older adults’ degree of depressive symptoms. As with general self-efficacy, where people with higher levels of self-efficacy tend to interpret environment demands as challenges rather than as threats (Bandura, 2001), it would be logical for older adults who consider themselves more effective at perceiving and using their emotions to feel more capable and motivated to struggle against the emotional distress generated by certain life events, thereby experiencing less depressive symptomatology. The mediational model also reveals the possible positive influence that ability EI may have on the levels of ESE, ensuring success in coping with emotional tasks which would lead to more frequent mastery experiences and less negative emotional arousal (Conger & Kanungo, 1988; Wise & Trunnell, 2001). In this sense, it is true that there are alternative explanations that speculate with the hypothesis of the influence of the ESE levels on ability EI (Alessandri, Vecchione & Caprara, 2015), which would have to be tested empirically. But even in that case, what is likely to occur it is a reciprocal effect between ability EI and ESE, thus requiring the application of longitudinal designs to check the possible reciprocal effects at different times.

Although previous work found that RESE predicted less depressive symptomatology (Extremera et al., 2006), the results obtained in relation to the ROE dimension were nonsignificant, contrary to the initial hypothesis. A possible explanation may be that older adults usually use emotion regulation strategies more focused on avoiding unpleasant situations than on changing emotional responses (see Márquez et al., 2004, for a review). Hence, when certain unpleasant life events are unavoidable, they have more difficulties to regulate their negative emotions and reduce the symptoms of depression. In addition, as expected, self-efficacy to perceive others’ emotions failed to predict symptoms of depression. This makes sense, as this interpersonal dimension of ESE assesses one’s perceived effectiveness to identify and address other people’s emotions, which seems irrelevant when addressing one’s own emotional discomfort in unpleasant or threatening situations. In fact, some authors suggest that this ESE dimension could be more useful in conflict situations and social interaction (Choi, Kluemper & Sauley, 2013), rather than to manage emotional states in oneself. In line with the findings of previous works (Fernández-Berrocal et al., 2005; Extremera et al., 2006; Goldenberg, Matheson & Mantler, 2006; Williams et al., 2009; Lloyd et al., 2012; Choi, Kluemper & Sauley, 2013), these results underscore the importance of intrapersonal ESE for mental health, revealing its relevance when addressing complex emotional events and protecting older adults from depression. In particular, the results suggest that older adults’ greater emotional competence generates a feeling of ESE, which in turn protects them from depressive symptoms.

On another hand, given that some studies have found sex differences, recording significantly higher levels of EI in women (Palmer et al., 2005; Extremera, Fernández-Berrocal & Salovey, 2006; McIntyre, 2010), greater female vulnerability to depression (Djernes, 2006, for a review), and some works have even found an EI-depression relationship only in men (Salguero, Extremera & Fernández-Berrocal, 2012), we decided to control the influence of sex as a covariate in the proposed mediational model. In the case of age, inconsistent results have usually been found in its relationship with EI (Fernández-Berrocal et al., 2012; Cabello et al., 2014; Fantini-Hauwel & Mikolajczak, 2014) and with depression (Snowdon, 2003), but we also decided to include it as a covariate. However, neither sex nor age proved to have a significant effect on the proposed mediational model between ability EI, ESE, and depressive symptomatology. A possible reason for this lack of relationship regarding sex could be that, among older adults like those of this sample, where the average age was about 80 years, the sex difference in the prevalence of depressive problems begins to be less pronounced (Baldwin, 1994). Another possible explanation is that institutionalization of these older adults in a residence may contribute to equating the cognitive functioning of both sexes. In this sense, admission into an institution is a traumatic event that requires the older adult to have high adaptation skills (Meléndez-Moral et al., 2013), and it can be a source of distress, provoking the onset of cognitive and emotional disorders (Riquelme, 1997), and depressive symptoms are frequent (Calkins & Cassella, 2007). However, it is still necessary to perform more research to shed light on these relationships. Additionally, in contrast to cognitive functioning, which has been shown to decline with age (Cabello et al., 2014), our results show that emotional functioning and depressive symptomatology are not significantly affected by age, suggesting the potential utility of ability EI and ESE in psychosocial interventions focused on this population group.

## Conclusions

The results of this study emphasize that EI and, especially, older adults’ beliefs about their efficacy to perceive and use their emotions are an important factor to predict levels of depressive symptoms. Thus, detecting reduced ESE could be an effective way to identify institutionalized older adults who are at risk of depression. This predictive capacity would be added to other variables more widely studied among residents, such as comorbidities with other diseases, pain, cognitive deficits, previous hospitalizations, or lack of social support (McCusker et al., 2013; Santiago & Mattos, 2014). The present results suggest that having adequate emotional ability, by itself, is not relevant to older adults’ psychological adjustment, but rather the increase in certain ESE beliefs it produces is the key variable capable of affecting depressive symptomatology.

Moreover, as some preliminary works with other groups have shown (Kotsou et al., 2011; Nelis et al., 2011), if older adults’ emotional competencies are trained, and this makes them feel emotionally effective, they will be able to perceive, use, understand and regulate their emotions more easily and adaptively, preventing depression and its symptoms. In particular, following the structure of effective intervention programs developed from the same theoretical perspective (Ruiz-Aranda et al., 2012; Rivers et al., 2013), it could be very beneficial to implement a program extended over time in which older adults become familiar with the four EI abilities, are trained in these skills through exercises of progressive complexity, and such learning is applied to everyday situations. This type of intervention would probably lead to greater ESE, a construct considered susceptible of improvement through practice and experience (Choi, Kluemper & Sauley, 2013), and would help older adults to feel that they have sufficient emotional resources to deal with emotionally demanding situations that may occur in residences.

Among the main limitations of this work is the fact that we used cross-sectional methodology with a relatively limited sample, which precludes the assumption of a causal relationship between the variables of study. For that reason, it would be interesting for future works—experimental, longitudinal, or through structural equation models (SEM)—to explore and confirm the causal relationships indicated by our results or even the presence of reciprocal effects between ability EI and ESE. In this regard, we recommend that future studies, for example, using SEM methodology, test more complex models in which the dimensions of ability EI could be analyzed separately in the EI-depression relationship, as well as the role of variables such as personality or intelligence. In addition, it would be useful if they could replicate the findings of this study using a wider sample and more objective measures of depression that are not exclusively based on participants’ self-reports. A final limitation of the study has to do with the use of WLEIS (Wong & Law, 2002), which does not cover all dimensions of ESE (not including self-perceived ability to understand emotions). Therefore, in future works it would be helpful to include specific scales of ESE (Choi, Kluemper & Sauley, 2013).

##  Supplemental Information

10.7717/peerj.2246/supp-1 Supplemental Information 1 Raw data The file contains data on participants’ responses to the study variables.

## Additional Information and Declarations

### Data Availability

The following information was supplied regarding data availability: The raw data has been supplied as Data S1.

## References

[ref-1] Alessandri G, Vecchione M, Caprara GV (2015). Assessment of regulatory emotional self-efficacy beliefs: a review of the status of the art and some suggestions to move the field forward. Journal of Psychoeducational Assessment.

[ref-2] American Psychiatric Association (2000). Diagnostic and statistical manual of mental disorders DSM-IV-TR.

[ref-3] Ashkanasy NM, Dasborough MD (2015). Reintroducing emotional intelligence: what it is and where we stand now. Emotion Researcher.

[ref-4] Baldwin R, Jacoby R, Oppenheimer C, Dening T, Thomas A (2008). Mood disorders: depressive disorders. *Oxford textbook of old age psychiatry*.

[ref-5] Baldwin RC (1994). Is there a distinct subtype of major depression in the elderly?. *Journal of Psychopharmacology*.

[ref-6] Bandura A (1977). Self-efficacy: toward a unifying theory of behavioral change. Psychological Review.

[ref-7] Bandura A (1986). Social foundations of thought and action: a social cognitive theory. Englewood Cliffs.

[ref-8] Bandura A (1997). *Self-efficacy: the exercise of control*.

[ref-9] Bandura A (2001). Social cognitive theory: an agentic perspective. Annual Review of Psychology.

[ref-10] Bandura A, Pajares F, Urdan T (2006). Guide for constructing self-efficacy scales. *Self-efficacy beliefs of adolescents*.

[ref-11] Bandura A, Adams NE, Beyer J (1977). Cognitive processes mediating behavioral change. Journal of Personality and Social Psychology.

[ref-12] Bandura A, Caprara GV, Barbaranelli C, Gerbino M, Pastorelli C (2003). Role of affective self-regulatory efficacy on diverse spheres of psychosocial functioning. Child Development.

[ref-13] Baron RM, Kenny DA (1986). The moderator–mediator variable distinction in social psychological research: conceptual, strategic, and statistical considerations. Journal of Personality and Social Psychology.

[ref-14] Boorsma M, Joling K, Dussel M, Ribbe M, Frijters D, Van Marwijk HWJ, Nijpels G, Van Hout H (2012). The incidence of depression and its risk factors in Dutch nursing homes and residential care homes. American Journal of Geriatric Psychiatry.

[ref-15] Brackett MA, Mayer JD (2003). Convergent, discriminant, and incremental validity of competing measures of emotional intelligence. Personality and Social Psychology Bulletin.

[ref-16] Brackett MA, Rivers SE, Shiffman S, Lerner N, Salovey P (2006). Relating emotional to social functioning: a comparison of self-report and performance measures of emotional intelligence. Journal of Personality and Social Psychology.

[ref-17] Brasseur S, Grégoire J, Bourdu R, Mikolajczak M (2013). The profile of emotional competence (PEC): development and validation of a self-reported measure that fits dimensions of emotional competence theory. PLoS ONE.

[ref-18] Cabello R, Navarro B, Latorre JM, Fernández-Berrocal P (2014). Ability of university-level education to prevent age-related decline in emotional intelligence. Frontiers in Aging Neuroscience.

[ref-19] Calkins M, Cassella C (2007). Exploring the cost and value of private versus shared bedrooms in nursing homes. The Gerontologist.

[ref-20] Caprara GV, Giunta LD, Eisenberg N, Gerbino M, Pastorelli C, Tramontano C (2008). Assessing regulatory emotional self-efficacy in three countries. Psychological Assessment.

[ref-21] Caprara GV, Steca P, Cervone D, Artistico D (2003). The contribution of self-efficacy beliefs to dispositional shyness: on social-cognitive systems and the development of personality dispositions. Journal of Personality.

[ref-22] Choi S, Kluemper DH, Sauley KS (2013). Assessing emotional self-efficacy: evaluating validity and dimensionality with cross-cultural samples. Applied Psychology.

[ref-23] Cobb CD, Mayer JD (2000). Emotional intelligence. Educational Leadership.

[ref-24] Conger JA, Kanungo RN (1988). The empowerment process: integrating theory and practice. Academy of Management Review.

[ref-25] Cutrona CE, Troutman BR (1986). Social support, infant temperament, and parenting self-efficacy: a mediational model of postpartum depression. Child Development.

[ref-26] Djernes JK (2006). Prevalence and predictors of depression in populations of elderly: a review. Acta Psychiatrica Scandinavica.

[ref-27] Ensinck KT, Schuurman AG, Van den Akker M, Metsemakers FM, Kester AD, Knottnerus JA, Buntinx F (2002). Is there an increased risk of dying after depression?. *American Journal of Epidemiology*.

[ref-28] Extremera N, Fernández-Berrocal P (2009). *Test de Inteligencia Emocional de Mayer, Salovey, Caruso*.

[ref-29] Extremera N, Fernández-Berrocal P, Ruiz-Aranda D, Cabello R (2006). Inteligencia emocional, estilos de respuesta y depresión. *Ansiedad y Estrés*.

[ref-30] Extremera N, Fernández-Berrocal P, Salovey P (2006). Spanish version of the Mayer-Salovey-Caruso Emotional Intelligence Test (MSCEIT), Version 2.0: Reliabilities, age and gender differences. *Psicothema*.

[ref-31] Fantini-Hauwel C, Mikolajczak M (2014). Factor structure, evolution, and predictive power of emotional competencies on physical and emotional health in the elderly. Journal of Aging and Health.

[ref-32] Fariselli L, Ghini M, Freedman J (2006). Age and emotional intelligence. Six Seconds.

[ref-33] Fernández-Berrocal P, Cabello R, Castillo R, Extremera N (2012). Gender differences in emotional intelligence: the mediating effect of age. Behavioral Psychology.

[ref-34] Fernández-Berrocal P, Extremera N (2009). La inteligencia emocional y el estudio de la felicidad. Revista *Interuniversitaria de Formación del Profesorado*.

[ref-35] Fernández-Berrocal P, Pérez JC, Repetto E, Extremera N (2004). April. Una comparación empírica entre cinco medidas breves de inteligencia emocional percibida.

[ref-36] Fernández-Berrocal P, Salovey P, Vera A, Extremera N, Ramos N (2005). Cultural influences on the relation between perceived emotional intelligence and depression. International Review of Social Psychology.

[ref-37] Fiori M, Antonietti J, Mikolajczak M, Luminet O, Hansenne M, Rossier J (2014). What is the ability emotional intelligence test (MSCEIT) good for? An evaluation using item response theory. PLoS ONE.

[ref-38] Folstein MF, Folstein SE, McHugh PR (1975). “Mini-mental state”. A practical method for grading the cognitive state of patients for the clinician. Journal of Psychiatric Research.

[ref-39] Frazier PA, Tix AP, Barron KE (2004). Testing moderator and mediator effects in counseling psychology research. Journal of Counseling Psychology.

[ref-40] Goldenberg I, Matheson K, Mantler J (2006). The assessment of emotional intelligence: a comparison of performance based and self report methodologies. Journal of Personality Assessment.

[ref-41] Hoyle RH, Kenny DA, Hoyle R (1999). Sample size, reliability, and tests of statistical mediation. *Statistical strategies for small sample research*.

[ref-42] Instituto Nacional de Estadística (2004). Censo de Población y Viviendas 2001. Resultados definitivos.

[ref-43] Instituto Nacional de Estadística (2013). Censo de Población y Viviendas 2011. Población residente en establecimientos colectivos.

[ref-44] Jongenelis K, Pot AM, Eisses AMH, Beekman ATF, Kluiter H, Ribbe MW (2004). Prevalence and risk indicators of depression in elderly nursing home patients: the AGEDstudy. *Journal of Affective Disorders*.

[ref-45] Jordan PJ, Troth AC, Ashkanasy NM, Burke RJ, Fox S, Cooper CL (2013). Emotional intelligence and human frailty at work: can we be too emotionally intelligent?. *Human frailties: wrong turns on the drive to success*.

[ref-46] Kafetsios K (2004). Attachment and emotional intelligence abilities across the life course. Personality and Individual Differences.

[ref-47] Katon WJ, Lin E, Russo J, Unützer J (2003). Increased medical costs of a population-based sample of depressed elderly patients. Archives of General Psychiatry.

[ref-48] Kirk BA, Schutte NH, Hine DW (2008). Development and preliminary validation of an emotional self-efficacy scale. Personality and Individual Differences.

[ref-49] Kotsou I, Nelis D, Grégoire J, Mikolajczak M (2011). Emotional plasticity: conditions and effects of improving emotional competence in adulthood. Journal of Applied Psychology.

[ref-50] Lloyd SJ, Malek-Ahmadi M, Barclay K, Fernandez MR, Chartr MS (2012). Emotional intelligence (EI) as a predictor of depression status in older adults. Archives of Gerontology and Geriatrics.

[ref-51] Lobo A, Ezquerra J, Gómez-Burgada F, Sala JM, Seva-Díaz A (1979). El “Mini-Examen Cognoscitivo”: un test sencillo, práctico, para detectar alteraciones intelectivas en pacientes médicos. Actas Luso Españolas de Neurología, Psiquiatría y Ciencias Afines.

[ref-52] Lopes PN, Salovey P, Straus R (2003). Emotional intelligence, personality, and the perceived quality of social relationships. Personality and Individual Differences.

[ref-53] MacKinnon DP, Lockwood CM, Williams J (2004). Confidence limits for the indirect effect: distribution of the product and resampling methods. Multivariate Behavioral Research.

[ref-54] Márquez M, Izal M, Montorio I, Pérez-Rojo G (2004). Emoción en la vejez: una revisión de la influencia de los factores emocionales sobre la calidad de la vida de las personas mayores. Revista Española de Geriatría y Gerontología.

[ref-55] Martins A, Ramalho N, Morin E (2010). A comprehensive meta-analysis of the relationship between emotional intelligence and health. Personality and Individual Differences.

[ref-56] Mayer JD, Roberts RD, Barsade S (2008). Human abilities: emotional intelligence. Annual Review of Psychology.

[ref-57] Mayer JD, Salovey P, Salovey P, Sluyter D (1997). What is emotional intelligence?. *Emotional development and emotional intelligence: implications for educators*.

[ref-58] Mayer JD, Salovey P, Caruso D (2002). *The Mayer-Salovey-Caruso Emotional Intelligence Test (MSCEIT)*.

[ref-59] McCusker J, Cole MG, Voyer P, Monette J, Champoux N, Ciampi A, Vu M, Dyachenko A, Belzile E (2013). Observed-rated depression in long-term care: frequency and risk factors. Archives of Gerontology and Geriatrics.

[ref-60] McIntyre MH (2010). Gender differences in the nature and linkage of higher-order personality factors to trait and ability emotional intelligence. Personality and Individual Differences.

[ref-61] Meléndez-Moral JC, Charco-Ruiz L, Mayordomo-Rodríguez T, Sales-Galán A (2013). Effects of a reminiscence program among institutionalized elderly adults. Psicothema.

[ref-62] Mikels JA, Löckenhoff CE, Maglio SJ, Goldstein MK, Garber A, Carstensen LL (2010). Following your heart or your head: focusing on emotions versus information differentially influences the decisions of younger and older adults. Journal of Experimental Psychology: Applied.

[ref-63] Mitchell AJ, Subramaniam H (2005). Prognosis of depression in old age compared to middle age: a systematic review of comparative studies. American Journal of Psychiatry.

[ref-64] Moussavi S, Chatterji S, Verdes E, Tandon A, Patel V, Utsum B (2007). Depression, chronic diseases, and decrements in health: results from the World Health Surveys. *The Lancet*.

[ref-65] Nelis D, Kotsou I, Quoidbach J, Hansenne M, Weytens F, Dupuis P, Mikolajczak M (2011). Increasing emotional competence improves psychological and physical wellbeing, social relationships, and employability. Emotion.

[ref-66] Palmer BR, Gignac G, Manocha R, Stough C (2005). A psychometric evaluation of the Mayer-Salovey-Caruso Emotional Intelligence Test version 2.0. Intelligence.

[ref-67] Preacher KJ, Hayes AF (2004). SPSS and SAS procedures for estimating indirect effects in simple mediation models. Behavior Research Methods, Instruments and Computers.

[ref-68] Riquelme A (1997). *Depresión en residencias geriátricas: un estudio empírico*.

[ref-69] Riquelme A, Buendía J, López A (2006). Desarrollo y validación de un instrumento para la evaluación de la depresión en ancianos. Psicothema.

[ref-70] Rivers SE, Brackett MA, Reyes MR, Elbertson NA, Salovey P (2013). Improving the social and emotional climate of classrooms: a clustered randomized controlled trial testing The RULER Approach. Prevention Science.

[ref-71] Ron P (2004). Depression, hopelessness, and suicidal ideation among the elderly: a comparison between men and women living in nursing homes and in the community. *Journal of Gerontological Social Work*.

[ref-72] Ruiz-Aranda D, Salguero JM, Cabello R, Palomera R (2012). Can an emotional intelligence program improve adolescents’ psychosocial adjustment? Results from The Intemo Project. Social Behavior and Personality.

[ref-73] Salguero JM, Extremera N, Cabello R, Fernández-Berrocal P (2015). If you have high Emotional Intelligence (EI), you must trust in your abilities: the interaction effect of ability EI and perceived EI on depression in women. Journal of Psychoeducational Assessment.

[ref-74] Salguero JM, Extremera N, Fernández-Berrocal P (2012). Emotional intelligence and depression: the moderator role of gender. Personality and Individual Differences.

[ref-75] Salovey P, Mayer JD (1990). Emotional Intelligence. Imagination, Cognition, and Personality.

[ref-76] Saltzman KM, Holahan CJ (2002). Social support, self-efficacy, and depressive symptoms: an integrative model. Journal of Social and Clinical Psychology.

[ref-77] Samanez-Larkin GR, Carstensen LL, Decety J, Cacioppo JT (2011). Socioemotional functioning and the aging brain. *The handbook of social neuroscience*.

[ref-78] Santiago LM, Mattos IE (2014). Depressive symptoms in institutionalized older adults. Revista de Saúde Pública.

[ref-79] Schutte NS, Malouff JM, Mohiyeddini C, Eysenck M, Bauer S (2013). Adaptive emotional functioning: a comprehensive model of emotional intelligence. *Handbook of psychology of emotions: recent theoretical perspectives and novel empirical findings*.

[ref-80] Schutte NS, Malouff JM, Thorsteinsson EB, Bhullar N, Rooke SE (2007). A meta-analytic investigation of the relationship between emotional intelligence and health. Personality and Individual Differences.

[ref-81] Scocco P, Rapattoni M, Fantoni G (2006). Nursing home institutionalization: a source of eustress or distress for the elderly?. *International Journal of Geriatric Psychiatry*.

[ref-82] Segulin N, Deponte A (2007). The evaluation of depression in the elderly: a modification of the Geriatric Depression Scale (GDS). Archives of Gerontology and Geriatrics.

[ref-83] Snowdon J, Sachdev PS (2003). Age variation in the prevalence of depression: are study findings meaningful?. *The Ageing Brain*.

[ref-84] Swan A, Watson HJ, Nathan PR (2009). Quality of life in depression: an important outcome measure in an outpatient cognitive-behavioural therapy group programme. Clinical Psychology and Psychotherapy.

[ref-85] Tombaugh TN, McIntyre NJ (1992). The Mini-Mental State Examination: a comprehensive review. Journal of the American Geriatric Society.

[ref-86] Vinyoles E, Vila J, Argimon JM, Espinas J, Abos T, Limón E (2002). Concordance among Mini-Examen Cognoscitivo and Mini-Mental State Examination in cognitive impairment screening Spanish. Atención Primaria.

[ref-87] Williams C, Daley D, Burnside E, Hammond-Rowley S (2009). Measuring emotional intelligence in preadolescence. Personality and Individual Differences.

[ref-88] Wise J, Trunnell P (2001). The influence of sources of self-efficacy upon efficacy strength. Journal of Sport & Exercise Psychology.

[ref-89] Wong CS, Law KS (2002). The effects of leader and follower emotional intelligence on performance and attitude: an exploratory study. The Leadership Quarterly.

[ref-90] World Health Organization (1992). The ICD-10 classification of mental and behavioural disorders: clinical descriptions and diagnosis guidelines.

